# Hepatitis B virus X protein downregulates expression of the miR-16 family in malignant hepatocytes *in vitro*

**DOI:** 10.1038/bjc.2011.190

**Published:** 2011-05-31

**Authors:** G Wu, F Yu, Z Xiao, K Xu, J Xu, W Tang, J Wang, E Song

**Affiliations:** 1 Department of Hepatobiliary Surgery, Sun-Yat-Sen Memorial Hospital, Sun-Yat-Sen University, 107 Yanjiang West Road, Guangzhou 510120, People's Republic of China; 2 Department of Breast Surgery, Sun-Yat-Sen Memorial Hospital, Sun-Yat-Sen University, 107 Yanjiang West Road, Guangzhou 510120, People's Republic of China

**Keywords:** HBx, miRNAs, miR-16 family, c-Myc, hepatocellular carcinoma

## Abstract

**Background:**

Hepatitis B virus X protein (HBx) is involved in the initiation and progression of hepatocellular carcinoma (HCC) by regulating the host protein-coding genes. In this study, we showed that HBx altered the expression of microRNAs (miRNAs) to promote proliferation and transformation in malignant hepatocytes *in vitro.*

**Methods:**

miRNA microarray and quantitative reverse-transcription polymerase chain reactions (qRT-PCRs) were performed to identify miRNAs that were differentially regulated by HBx in HCC cells. Protein, mRNA, and miRNA expression analyses; cell cycle and apoptosis analyses; loss/gain-of-function analysis; and luciferase reporter assays were performed to delineate the consequences of miR-16 family repression in HepG2 cells.

**Results:**

Hepatitis B virus X protein induced widespread deregulation of miRNAs in HepG2 cells, and the downregulation of the miR-16 family was reproducible in HepG2, SK-HEP-1, and Huh7 cells. *CCND1*, a target of the miR-16 family, was derepressed by HBx in HepG2 cells. c-Myc mediated the HBx-induced repression of miR-15a/16 in HepG2 cells. Ectopically expressed miR-15a/16 suppressed the proliferation, clonogenicity, and anchorage-independent growth of HBx-expressing HepG2 cells by arresting them in the G1 phase and inducing apoptosis, whereas reduced expression of miR-16 accelerated the growth and cell-cycle progression of HepG2 cells.

**Conclusions:**

Hepatitis B virus X protein altered the *in vitro* expression of miRNAs in host malignant hepatocytes, particularly downregulating the miR-16 family. Repression of miR-15a/16 is c-Myc mediated and is required for the HBx-induced transformation of HepG2 cells *in vitro*. Therefore, miR-16 family may serve as a therapeutic target for hepatitis B virus (HBV)-associated HCC.

Chronic hepatitis B virus (HBV) infection is the leading cause of hepatocellular carcinoma (HCC) in Asia and West Africa (El-Serag and Rudolph, 2007). In addition, HBV-infected HCCs exhibit more aggressive biological characteristics during clinical disease progression than do non-infected tumours. Hepatitis B virus X protein (HBx), an oncoprotein encoded by HBV, has been confirmed to have a role in HBV-related hepatocarcinogenesis (Arbuthnot *et al*, 2000). It is a transactivating protein that alters host gene expression by constitutively activating cytoplasmic signal transduction pathways (e.g., NF-*κ*B, Src, Ras, AP-1, AP-2, PI3K/Akt, Jak/STAT, Smad, and Wnt) and by binding to nuclear transcription factors (e.g., CREB, ATF-2, Oct-1, TBP, and basal transcription factors), contributing to increased cell proliferation and survival (Feitelson and Lee, 2007). It has been reported that HBx expression promotes HCC in mice (Kim *et al*, 1991; Terradillos *et al*, 1997) and induces hepatocyte transformation in culture (Seifer *et al*, 1991).

Increasing evidence has demonstrated that non-coding RNAs (ncRNAs) are a new class of functional transcripts in eukaryotic cells (Costa, 2005), including microRNAs (miRNAs), which are 21- to 23-nucleotide RNA molecules that regulate the stability or translational efficiency of target mRNAs (Bartel, 2009). Primary miRNA transcripts (pri-miRNAs) are sequentially processed by two RNase III enzymes, Drosha and Dicer, to yield mature miRNAs (Kim, 2005). microRNAs are deregulated in many human diseases, especially in tumourigenesis. The pattern of miRNA expression can be correlated with cancer type, stage, and other clinical variables. microRNA expression analyses have also suggested oncogenic (or tumour-suppressive) roles of miRNAs. Of note, miRNAs have roles in almost all aspects of cancer biology, such as proliferation, apoptosis, invasion/metastasis, and angiogenesis (Lee and Dutta, 2009). The location of miRNAs in cancer-associated genomic regions, epigenetic mechanisms, and alterations in the miRNA processing machinery all contribute to the widespread differential expression of miRNA genes in malignant cells compared with normal cells (Calin and Croce, 2006).

Recently, miRNAs have been confirmed to function in viral pathogenesis by regulating the expression of genes from both the viral and host genomes. Epstein-Barr virus (EBV) encodes its own miRNAs, whereas its oncoprotein, LMP-1, induces the expression of host miR-146a and miR-155 via activation of NF-*κ*B to modulate the innate immune response to the virus-infected host cell (Motsch *et al*, 2007). Similarly, human T-lymphotropic virus type I (HTLV-1) transforms host cells by inducing dysregulation of the expression of specific miRNAs, including miR-146a, which is upregulated by Tax (an oncoprotein of HTLV-1) (Pichler *et al*, 2008). Furthermore, miR-193b expression is induced by the hepatitis C virus (HCV), and this expression facilitates chemosensitivity to sorafenib in malignant hepatocytes (Braconi *et al*, 2010).

In this study, we first hypothesised that HBx expression in hepatocytes would alter host cellular miRNA expression, leading to HCC initiation and progression. We identified the significantly changed miRNAs (>2-fold) between HepG2-hbx and HepG2-vc cells via microarray. The expression of HBx caused widespread miRNA repression in HepG2 cells, including the miR-16 family. Members of this family are frequently downregulated in multiple haematopoietic and solid tumours, including HCC (Huang *et al*, 2009), and this prompted us to further research regarding their repression. Downregulation of the miR-16 family was validated by quantitative reverse-transcription polymerase chain reaction (qRT-PCR) analysis of the HepG2, Huh7, and SK-HEP-1 cells that expressed HBx. According to these results, *CCND1*, a target of the miR-16 family, was derepressed in HepG2-hbx cells. Next, we confirmed that the induction of host c-Myc mediated the suppression of miR-15a/16 in HepG2-hbx cells. Finally, we found that ectopically expressed miR-15a/16 suppressed the proliferation, clonogenicity, and anchorage-independent growth of HBx-expressing HepG2 cells by arresting cells in the G1 phase and inducing apoptosis, while the miR-16 inhibitor promoted the growth and cell-cycle progression of HepG2 cells.

## Materials and methods

### Cell culture

The human HepG2, SK-HEP-1, and Huh7 HCC cell lines were purchased from American Type Culture Collection (ATCC, Manassas, VA, USA) and cultured in Dulbecco's modification of Eagle's medium (DMEM; Gibco, Carlsbad, CA, USA) supplemented with 10% fetal bovine serum (FBS; HyClone, South Logan, UT, USA). The cells were cultured in DMEM supplemented with 10% FBS and 500 ng ml^−1^ G418 (Invitrogen, Carlsbad, CA, USA) (HepG2-hbx/HepG2-2.1, cells stably expressing HBx protein and HepG2-vc, cells stably transfected with PCDNA3.1 plasmid) or 380 ng ml^−1^ G418 (HepG2.2.15, HepG2 cells stably transfected with the entire HBV genome). All cell lines were maintained in a humidified atmosphere with 5% CO_2_.

### RNA oligoribonucleotides and plasmids

The HBx open reading frame (Adr subtype, AB299858) was cloned into the PCDNA3.1 (+) plasmid (Invitrogen) to create PCDNA3.1-hbx. All RNA oligoribonucleotides, including miR-15a/16 mimics/inhibitors, c-Myc-specific siRNA, and negative control small RNAs (NC), were purchased from GenePharma (Shanghai, China).

### Cell transfection

RNA oligoribonucleotides and plasmids were transfected using Lipofectamine 2000 (Invitrogen), according to the manufacturer's instructions. 200 nM of miR-16 inhibitor and inhibitor NC, or 50 nM of other RNA oligoribonucleotides was used in cell transfection. For stable transfection, HepG2 cells were exposed to 900 ng ml^−1^ G418 24 h after transfection with PCDNA3.1-hbx or PCDNA3.1. G418-resistant cells were further cultured to select the HBx mRNA- and protein-expressing monoclones.

### Reverse-transcription polymerase chain reaction and qRT-PCR

Total RNA was extracted with TRIzol reagent (Invitrogen) and treated with DNase I (Tiangen Biotech, Beijing, China). cDNA for reverse-transcription polymerase chain reaction (RT-PCR) was synthesised with the PrimeScript RT-PCR kit (Takara Biotechnology, Dalian, China). The PCR amplification procedure for *β*-actin was as follows: 94°C for 3 min; 30 cycles of 94°C for 40 s, 56°C for 45 s, and 72°C for 60 s; followed by terminal elongation at 72°C for 7 min. The amplification of HBx was performed as follows: 95°C for 2 min; 30 cycles of 95°C for 30 s, 62.5°C for 30 s, and 72°C for 30 s; followed by 72°C for 5 min. The qRT–PCR assays were performed using the SYBR PrimeScript RT-PCR kit (Takara Biotechnology). The assay kits for U6, miR-27a, miR-21, miR-663, miR-15a, miR-15b, and miR-16 were purchased from GenePharma; the TaqMan miRNA assay kit for miR-16 was purchased from Ambion (Foster City, CA, USA). snoRNAs, including RNU48, RNU44, U47, and RNU6B, were also purchased from Ambion and used as endogenous controls.

### Immunoblots

Cell cytosolic protein fractions were prepared using RIPA buffer (Beyotime Biotechnology, Haimen, China). The antibodies used in western blotting were HBx (sc-71239; Santa Cruz Biotechnology, Santa Cruz, CA, USA), *β*-actin (sc-130301; Santa Cruz), c-Myc (9402; Cell Signaling Technology (CST), Boston, MA, USA), CCND1 (2926; CST), and GAPDH (BA2913; Wuhan Boster Biological Technology, Wuhan, China).

### miRNA microarrays

The method for the miRNA microarray analysis was described in our previous report, except for the addition of an updated miRNA probe set (the NCode Human miRNA Microarray Probe Set V3+, Invitrogen) composed of 703 known and 393 predicted human miRNAs in the present work (Yu *et al*, 2007).

### Cell viability and apoptosis analysis

Cell proliferation was analysed with the Cell Counting Kit-8 (CCK-8; Dojindo Laboratories, Kumamoto, Japan). The sphere formation assay was performed by culturing the cells (1000 cells ml^−1^) in suspension in serum-free DMEM-F12 supplemented with B27 (1 : 50, Invitrogen), 20 ng ml^−1^ EGF (BD Biosciences, San Jose, CA, USA), 0.4% bovine serum albumin (Sigma, St Louis, MO, USA), and 4 mg ml^−1^ insulin (Sigma). For the clonogenicity analysis, 24 h after transfection, aliquots of 500 viable HepG2-hbx cells or 1000 viable HepG2.2.15 cells were placed in 6-cm dishes and maintained in 10 ml of complete medium for 2–3 weeks. Cell apoptosis was determined 48 h after the NC and miR-15a/16 mimic transfections using an Annexin V-FITC kit (Bender MedSystems, Vienna, Austria) and analysed using a BD FACSCalibur flow cytometer (Becton Dickinson, San Jose, CA, USA).

### Cell-cycle analysis

Twenty-four hours after the miRNA mimic and NC transfections, the cells were treated with nocodazole (100 ng ml^−1^, M1404; Sigma) for 20 h. Floating and adherent cells were harvested, combined, washed once in phosphate-buffered saline, and fixed in 70% ethanol overnight. The DNA content was then examined using the Cell Cycle Detection Kit (KGA512; KeyGen Biotechnology, Nanjing, China) and analysed using BD FACSCalibur flow cytometer.

### Luciferase reporter assay

PGL3cm-CCND1-3′UTR wild-type and pGL3cm-CCND1-3′UTR mutant vectors were received as generous gifts from Dr Shi-Mei Zhuang (School of Life Sciences, Sun-Yat-sen University) and were constructed as previously described (Xu *et al*, 2009). HepG2-hbx and HepG2-vc cells were grown in 48-well plates and were co-transfected with 2 ng of pRL-TK (Promega, Madison, WI, USA) and 5 ng of wild-type or mutant luciferase reporter. Forty-eight hours after transfection, the cells were harvested and subjected to a luciferase assay. The firefly luciferase activity was normalised to the Renilla luciferase activity.

The sequence of RNA and DNA oligoribonucleotides used can be found in Supplementary Table 1.

## Results

### Establishing the HepG2-hbx, HepG2-2.1 and HepG2-vc cell lines

**Figure 1:**
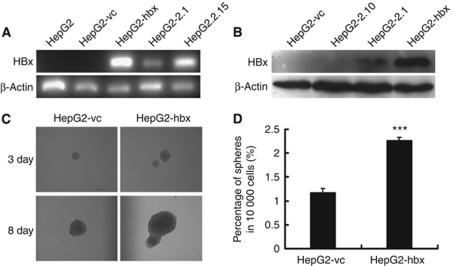
Generation of HepG2 cell lines stably expressing the HBx gene. (**A**) RT–PCR and (**B**) western blot analysis confirmed the mRNA and protein expression, respectively, of the HBx gene in the HepG2-hbx and HepG2-2.1 cell lines. HepG2 cells stably transfected with vector alone (HepG2-vc) were used as a negative control. (**C**, **D**) HepG2-hbx cells produced significantly larger and more numerous spheres in an anchorage-independent sphere formation assay than did HepG2-vc under serum-free conditions. Representative graphs are presented. The data are presented as mean numbers of spheres ±s.e. (^***^P<0.001; Student's *t*-test, *n*=3).

To investigate the impact of HBx on host miRNA expression in HepG2 cells, we made stably expressing clones. We transfected the HBx-expressing plasmid (HepG2-hbx or HepG2-2.1) or empty vector (HepG2-vc) into the HepG2 cells and then performed G418 selection. Clones including HepG2-2.10 were screened for HBx mRNA and protein expression (Figures 1A and B). Over time, the expression of HBx increased the size and number of anchorage-independent colonies in the HepG2-hbx cells to a greater degree than in the HepG2-vc cells (Figures 1C and D).

### Members of the miR-16 family were frequently suppressed by HBx expression in HepG2, Huh7, and SK-HEP-1 cells

The miRNA microarrays were analysed to identify the HBx-deregulated miRNAs in HepG2 cells. The results showed that of the 1096 miRNAs included in the assay, HBx caused widespread suppressions of miRNA expression, including the miR-16 family, in the HepG2-hbx cells compared with the HepG2-vc cells (>2-fold). Interestingly, few miRNAs, including miR-21, were upregulated by HBx expression. Therefore, further studies will be necessary to determine whether HBx impairs the biogenesis of miRNAs in HepG2 cells (Kumar *et al*, 2007). The detailed results of the microarray analysis are provided in Supplementary Table 2.

**Figure 2:**
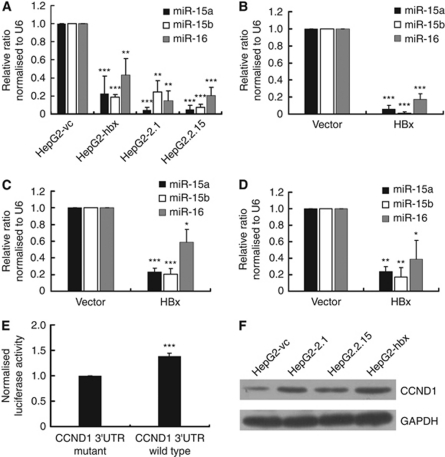
HBx downregulates the expression of the miR-16 family in non-HBV-infected malignant hepatocytes *in vitro* and upregulates the expression of *CCND1* in HepG2 cells. (**A**) The expression of the miR-16 family was measured using qRT-PCR and normalised by U6 expression in HepG2 cells that stably expressed HBx or control vector. (**B**–**D**) The expression of the miR-16 family normalised to U6 was detected by qRT-PCR in (**B**) HepG2, (**C**) Huh7, and (**D**) SK-HEP-1 cells transiently transfected with HBx-expressing plasmid or control vehicle. Each cell line was transfected with 4 *μ*g PCDNA3.1-hbx or 4 *μ*g PCDNA3.1 as a control. Cells were collected for analysis 48 h after each transfection. (**E**) The activity of the luciferase reporter containing the wild-type 3′UTR of *CCND1* was elevated in HepG2-hbx cells. pRL-TK was co-transfected with a firefly luciferase reporter plasmid carrying either the wild-type (WT) or mutant (MUT) 3′UTR of *CCND1* into HepG2-hbx or HepG2-vc cells; luciferase activity was analysed 48 h later. pRL-TK expressing Renilla luciferase was used to correct for the differences in transfection and harvest efficiencies between the HepG2-hbx and HepG2-vc cells. The ratio of the luciferase activity of MUT-3′UTR in HepG2-hbx to that in HepG2-vc cells was normalised to 1. (**F**) A western blot confirmed the induction of CCND1 by HBx in HepG2 cells. The data are presented as mean±s.e. fold. (^*^P<0.05, ^**^P<0.01, ^***^P<0.001; Student's *t*-test, NPar tests; *n*=3).

Because the miR-16 family has been reported to have multiple roles in tumour suppression (Bonci *et al*, 2008), we used qRT-PCR analysis to examine the expression of miR-16 family members in HepG2 cells that had been stably or transiently transfected with HBx. We found that the expression of these genes was significantly suppressed in both stably and transiently transfected cells (Figures 2A and B). To eliminate the deviation induced by using RNU6B as endogenous control, we further validated the HBx-mediated downregulation of miR-16 in HepG2 cells using the TaqMan miRNA assay, which was normalised to the means of the endogenous controls, including RNU48, RNU44, U47, and RNU6B (Supplementary Figure 1). To explore whether the HBx-mediated repression of the miR-16 family was reproducible or restricted to HepG2 cells, we transiently transfected PCDNA3.1-hbx and the control vector into non-HBV-infected Huh7 and SK-HEP-1 cells. Significantly, HBx also reduced the expression of the miR-16 family in both cell lines (Figures 2C and D). In confirmation of the microarray results, we successfully detected the HBx-induced downregulation of miR-27a/miR-663 and the upregulation of miR-21 in HepG2 cells (Supplementary Figure 2).

To analyse whether the effects of HBx could be detected as far downstream as the target genes of miR-16 family members, we examined the expression of *CCND1*, a reported target of the miR-16 family, which functions in the G1/S transition of the cell cycle. Using a luciferase assay, we demonstrated that HBx significantly upregulated the activity of the *CCND1* 3′UTR in HepG2-hbx compared with HepG2-vc cells (38±5% Figure 2E). Western blot results also confirmed the upregulation of CCND1 in HBx-expressing HepG2 cells (Figure 2F). These results were in accordance with reports that HBx stimulated cell-cycle progression (Benn and Schneider, 1995) and activated the expression of *CCND1* (Klein *et al*, 2003; Park *et al*, 2006; Jung *et al*, 2007).

In addition, we found that miR-16 exhibited an inverse upregulation when HBx achieved pro-apoptotic doses during transient transfection (Supplementary Figure 3). More apoptotic cells appeared when we increased the dose of PCDNA3.1-hbx in HepG2, Huh7, and SK-HEP-1 cells (data not shown). This finding may be attributed to the pro-apoptotic functions of both HBx (Terradillos *et al*, 1998; Arbuthnot *et al*, 2000) and miR-16 via the targeting of BCL-2 (Cimmino *et al*, 2005). More studies are needed to clarify the roles played by miR-16 in HBx-induced hepatocyte apoptosis (or the inhibition of apoptosis) and acute/chronic HBV infection.

Taken together, these results show that HBx causes global miRNA deregulation in HepG2 cells and that the miR-16 family is frequently repressed by HBx expression in HepG2, Huh7, and SK-HEP-1 cells. Accordingly, *CCND1*, a target of the miR-16 family, is derepressed by HBx in HepG2 cells.

### Loss-of-function of c-Myc restored the expression of miR-15a/16 in HepG2-hbx cells

**Figure 3:**
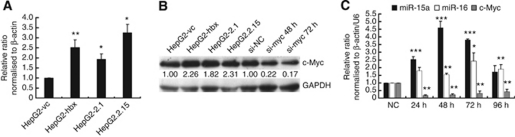
HBx suppresses the expression of miR-15a/16 by activating c-Myc in HepG2 cells. (**A**) qRT-PCR and (**B**) representative western blot results showed that HBx upregulated c-Myc transcription and translation in HepG2 cells. Relative c-Myc expression was normalised to *β*-actin and GAPDH. The qRT-PCR data are presented as mean±s.e. fold compared with HepG2-vc (*n*=3, Student's *t*-test). The c-Myc/GAPDH ratio is listed for the western blots. (**C**) The relative expression of c-Myc and miR-15a/16 was analysed by qRT-PCR at 24, 48, 72, and 96 h after the transfection of si-c-Myc into HepG2-hbx cells compared with the negative control (NC) siRNA transfectants, normalised to *β*-actin and U6, respectively. The data are presented as mean fold change±s.e. (^*^P< 0.05, ^**^P<0.01, ^***^P<0.001; *n*=3, Student's *t*-test).

To elucidate the underlying mechanisms of HBx-induced miR-16 family downregulation, we referred to a previous report showed that c-Myc repressed the promoter activity of pri-miR-15a/16-1 in B-cell lymphomas (Chang *et al*, 2008). We examined the expression of c-Myc in HepG2 cell lines stably transfected with HBx. Consistent with others’ results (Balsano *et al*, 1991; Kalra and Kumar, 2006; Li *et al*, 2010), HBx significantly upregulated the mRNA and protein expression of c-Myc in HepG2 cells (Figures 3A and B). As anticipated, the knock-down of c-Myc with a specific siRNA produced a remarkable rescue of the expression of miR-15a and miR-16 in HepG2-hbx cells at the indicated time points (Figure 3C). Of note, miR-15a was more sensitive to si-c-Myc treatment than was miR-16, which suggested that other mechanisms may be involved in the regulation of miR-16. This restoration could be reproduced in HepG2.2.15 cells (data not shown). These results suggest that HBx downregulates miR-15a/16 expression, at least partly, through the induction of c-Myc in HepG2 cells.

### Reduced expression of miR-16 promoted the growth and cell-cycle progression of HepG2 cells

**Figure 4:**
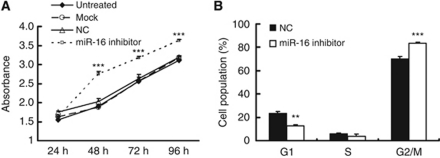
The miR-16 inhibitor promoted proliferation and cell-cycle progression in HepG2 cells. (**A**) The growth curves of HepG2 cells transfected with miR-16 inhibitor or NC were generated using the absorbance from the CCK-8 analysis at the indicated time points. The reduced expression of miR-16 significantly promoted the growth of HepG2 cells at 48, 72, and 96 h post-transfection. (**B**) HepG2 cells were treated with nocodazole (100 ng ml^−1^) 24 h after the transfection of miR-16 inhibitor or NC, and cell-cycle distribution was analysed 20 h later. The data are presented as mean fold change±s.e. (^**^P<0.01, ^***^P<0.001; *n*=3, Student's *t*-test).

Although the tumour-suppressive capacity of the miR-16 family has been well documented in other cancers, a loss-of-function assay was performed to determine the consequences of such repression in HepG2 cells. We knocked down miR-16 expression in HepG2 cells using a specific antisense inhibitor, and CCK-8 analysis showed that this miR-16 inhibitor significantly promoted cell proliferation at 48, 72, and 96 h post-transfection compared with the NC transfectants (Figure 4A). miR-16 inhibition also accelerated the G1/S transition in HepG2 cells (Figure 4B). The efficiency of the inhibitor was checked by qRT-PCR analysis (Supplementary Figure 4).

### Ectopically expressed miR-15a/16 suppressed the proliferation, clonogenicity, and anchorage-independent growth of HBx-expressing HepG2 cells by arresting cells at the G1 phase and inducing apoptosis

**Figure 5:**
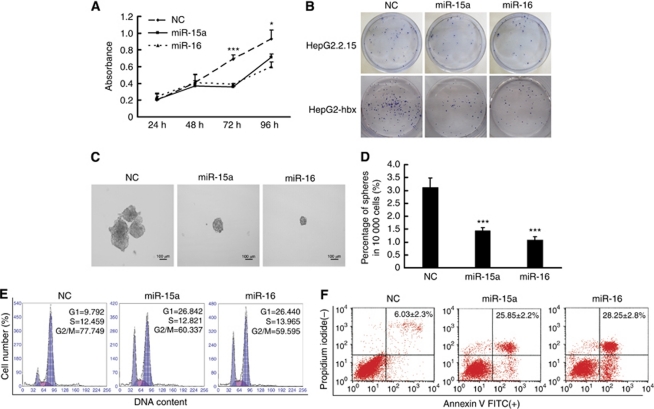
Ectopically expressed miR-15a/16 repressed the proliferation, clonogenicity, and anchorage-independent growth of HBx-transfected HepG2 cells *in vitro* by blocking cell-cycle progression and inducing apoptosis. (**A**) CCK-8 analysis showed that the expression of both miR-15a and miR-16 significantly suppressed the growth of HepG2-hbx cells at 72 and 96 h post-transfection. The data are presented as mean absorbance±s.e. (^*^P<0.05, ^***^P<0.001, *n*=3; Student's *t*-test). (**B**) HepG2-hbx and HepG2.2.15 cells transfected with miR-15a/16 or NC were grown at an extremely low density (e.g., 500–1000 cells in 10 ml of medium) for 2–3 weeks, and the clones were fixed with methanol and stained with 0.1% crystal violet in 20% methanol. Representative plates are presented. (**C** and **D**) HepG2-hbx cells (10 000) transfected with miR-15a/16 mimic or NC were placed in a 10-ml sphere formation assay-conditioned medium to grow anchorage independently for 10 days. The data are presented as the mean numbers of spheres ±s.e. (^***^P<0.001; *n*=3, Student's *t*-test). (**E**) HepG2-hbx cells were treated with nocodazole (100 ng ml^−1^) 24 h post-transfection, and the DNA ploidy was analysed 20 h later. 2N, cells containing diploid DNA; 4N, cells containing tetraploid DNA. (**F**) PI-Annexin V analysis was performed to detect the early apoptosis of HepG2-hbx cells transfected with miR-15a/16 or NC (48 h). The data are presented as mean±s.e. (*n*=3).

To inspect the therapeutic potential of the miR-16 family in HBV^+^ HCC, a series of *in vitro* experiments were performed. CCK-8 analysis showed that miR-15a/16 mimics repressed the growth of HepG2-hbx cells at 72 and 96 h post-transfection compared with NC-transfected cells (Figure 5A). Subsequently, HepG2-hbx and HepG2.2.15 cells were transfected with NC or miR-15a/16 mimics and were allowed to form foci at a low density. Notably, fewer colonies were seen in miR-15a/16 mimic-transfected HepG2-hbx and HepG2.2.15 cells compared with NC transfectants (Figure 5B). We further analysed the effects of miR-15a/16 on anchorage-independent growth in a serum-free medium. The results showed that miR-15a/16 expression in HepG2-hbx cells significantly reduced the size and number of the spheres compared with the NC transfectants (Figures 5C and D).

Next, we explored the mechanisms of the reduced survival of HepG2-hbx cells treated with miR-15a/16 mimics. Fluorescence-activated cell sorting results showed that the enforced expression of miR-15a/16 caused an accumulation of cells during the G1 phase in HepG2-hbx cells (Figure 5E). Moreover, miR-15a/16 mimics also induced significantly more apoptotic cells than NC transfectants in HepG2-hbx cells (Figure 5F). Collectively, these results illustrate that the miR-16 family could efficiently repress the proliferation and viability of HepG2-hbx cells by blocking cell-cycle progression and inducing apoptosis.

## Discussion

Hepatitis B virus X protein alters the expression of the host protein-coding genes by its transactivating function, thus contributing to the initiation and progression of HCC. However, most human genome transcripts are ncRNAs, including miRNAs, small RNAs, and long ncRNAs, all of which have been confirmed to be capable of regulating gene expression. The dysregulation of miRNAs and long ncRNAs is extensively involved in many human disease processes, including tumourigenesis (Matouk *et al*, 2007; Mercer *et al*, 2009; Wilusz *et al*, 2009; Gupta *et al*, 2010). At present, there have been few publications regarding the associations between HBx and host cellular miRNAs. In this study, we demonstrated that HBx altered host miRNA expression in malignant hepatocytes *in vitro*, including the c-Myc-mediated downregulation of miR-15a/16. Loss- and gain-of-function assays indicated the significance of miR-16 family deregulation in HepG2 cells transformed *in vitro*.

Hepatitis B virus X protein was found to cause widespread suppression of miRNAs expression, including the miR-16 family, in HepG2 cells, but the upregulation of only a few miRNAs. This miRNA expression pattern is considered to be a specific characteristic of tumours (Bottoni *et al*, 2005; Calin and Croce, 2006; Kumar *et al*, 2007). The downregulation of miR-15a, -16, -338, and -422a has also been reported by Liu *et al* (2009). They identified HBV-associated miRNAs differentially expressed between HepG2.2.15 and the parental HepG2 cells using microarrays and northern blot analyses. In the present study, we directly transfected HBx into HepG2 cells to establish clones that stably expressed HBx and demonstrated that the expression of the miR-16 family was downregulated in HepG2 cells and HepG2.2.15 cells (Figure 2A). However, HepG2.2.15, which is a HepG2 cell line transfected with a plasmid carrying four 5′–3′ tandem copies of the HBV genome, still did not completely simulate natural HBV infection, as multiple copies of HBV DNA were integrated into the stably transfected line (Sells *et al*, 1987). This is likely to influence cell function, and the role of the miR-16 family in HBV infection *in vivo* remains to be further investigated.

The miR-16 family is composed of miR-15a, -15b, and -16. The miR-15a/16-1 and miR-15b/16-2 gene clusters are located on human chromosomes 13q and 3 and are co-transcribed with *DLEU2* and *SMC4*, respectively (Yue and Tigyi, 2010). This family generally functions as a key regulator of the G1/S cell-cycle checkpoint by targeting *CCND1-3*, *CCNE1,* and *CDK6* (Linsley *et al*, 2007; Liu *et al*, 2008). Accordingly, the deletion of 13q (the location of miR-15a/16-1) is associated with the deregulation of genes involved in the cell cycle and proliferation in dedifferentiated HCCs (Skawran *et al*, 2008) and is also related to aggressive HCC behaviours (Wong *et al*, 2002). This family has also been shown to be repressed and to suppress (in specific tumour types) a series of oncogenes that include *c-Myb, Bmi-1, BCL-2, WNT3A,* and *Wip1* (Bottoni *et al*, 2005; Cimmino *et al*, 2005; Bonci *et al*, 2008; Bandi *et al*, 2009; Bhattacharya *et al*, 2009; Zhao *et al*, 2009; Zhu *et al*, 2009; Klein *et al*, 2010; Meyer-Rochow *et al*, 2010; Zhang *et al*, 2010). Moreover, members of the miR-16 family are involved in regulating chemosensitivity (Blower *et al*, 2008; Xia *et al*, 2008). With respect to HCC, the downregulation of miR-15a and miR-16-1 is a valuable predictor of HCC venous metastasis and patient survival (Budhu *et al*, 2008). High expression of miR-15b is correlated with a low risk of tumour recurrence following hepatectomy (Chung *et al*, 2010). In addition to hepatocytes, miR-15b and miR-16 are repressed during the activation of rat hepatic stellate cells (HSCs; Guo *et al*, 2009b), and miR-16 inhibits proliferation while increases the apoptosis of HSCs (Guo *et al*, 2009a). Importantly, HBx can induce paracrine activation and proliferation of HSCs in both human and rat liver via TGF-*β* (Martin-Vilchez *et al*, 2008). Collectively, these data suggest that the suppression of the miR-16 family contributes to the initiation (i.e., liver fibrosis/cirrhosis) and progression of HBV/HBx-induced HCCs.

The miR-16 family is the downstream target of several oncogenic and tumour suppressor networks. p53 facilitates the maturation of the miR-15a/16-1 cluster (Suzuki *et al*, 2009), and the expression of miR-15b/16-2 cluster is directly induced by E2F1 and E2F3 during the G1/S transition (Bueno *et al*, 2010). Radiation also induces the upregulation of miR-15a/16 in glioblastoma cells (Chaudhry *et al*, 2010) and in human endothelial cells (Wagner-Ecker *et al*, 2010). In contrast, the expression of the miR-16 family has been shown to be suppressed >1.5-fold by c-Myc and lin28B in B-cell lymphomas (Chang *et al*, 2008, 2009), which mirrors the preliminary HBx-c-Myc-miR-15a/16 pathway found in our study. c-Myc has been suggested to have a central role during the malignant transition in human hepatocarcinogenesis (Kaposi-Novak *et al*, 2009). Additionally, HER2 Delta 16 suppresses miR-15a/16 and deregulates BCL-2 to promote endocrine resistance in breast tumours (Cittelly *et al*, 2010), whereas PKC alpha downregulates miR-15a in head and neck squamous cell carcinoma (Cohen *et al*, 2009). Notably, because HBx can antagonise the function of p53 by itself (Dewantoro *et al*, 2006) or by recruiting c-Myc (Lee and Rho, 2000), we propose that HBx may induce c-Myc to repress the transcription and p53-mediated post-transcriptional maturation of miR-15a/16.

While this manuscript was being prepared, Wang *et al* (2010) reported for the first time that HBx induced the deregulation of cellular miRNAs in HepG2 cells and that the *let-7* family was downregulated in both HBx-transfected cell lines and HBV-infected HCC tumour tissue (Wang *et al*, 2010). The miR-16 family was not included in their microarray analysis; however, the repression of *let-7d* (>2-fold) was detected in our results. The disparities between these data may have resulted from differences in the HBx expression system (i.e., Wang *et al* employed a transient recombinant adenovirus infection, whereas we used stable transfection), differences in microarray sensitivity, and in the intensity of HBx protein expression.

In conclusion, we found that HBx altered the expression of cellular miRNAs in host malignant hepatocytes *in vitro*, including the repression of the miR-16 family. Furthermore, HBx-induced downregulation of miR-15a/16 in HepG2 cells was c-Myc mediated, while ectopically expressed miR-15a/16 repressed the proliferation, clonogenicity, and anchorage-independent growth of HepG2-hbx cells by inducing cell-cycle arrest and apoptosis. Our results highlight the therapeutic roles that targeting c-Myc and the miR-16 family may play in HBV-related chronic liver diseases.
