# Supplementary figures and images for: Hepatitis B virus X protein downregulates expression of the miR-16 family in malignant hepatocytes in vitro
Source: Br J Cancer. 2011 May 31;105(1):146–53. doi: 10.1038/bjc.2011.190 (PMC3137408; doi:10.1038/bjc.2011.190)

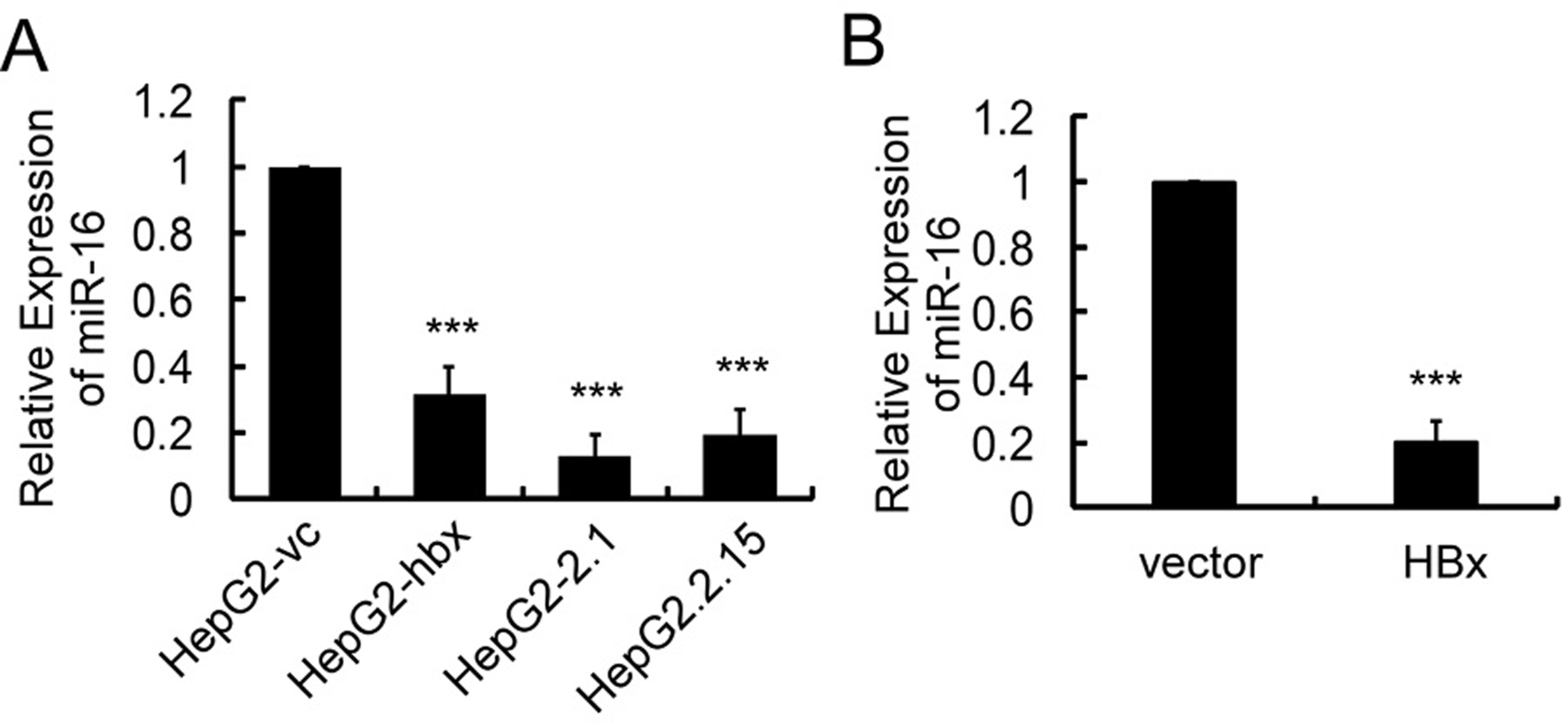

Supplement: Supplementary Figure 1 [file bjc2011190x1.tif]

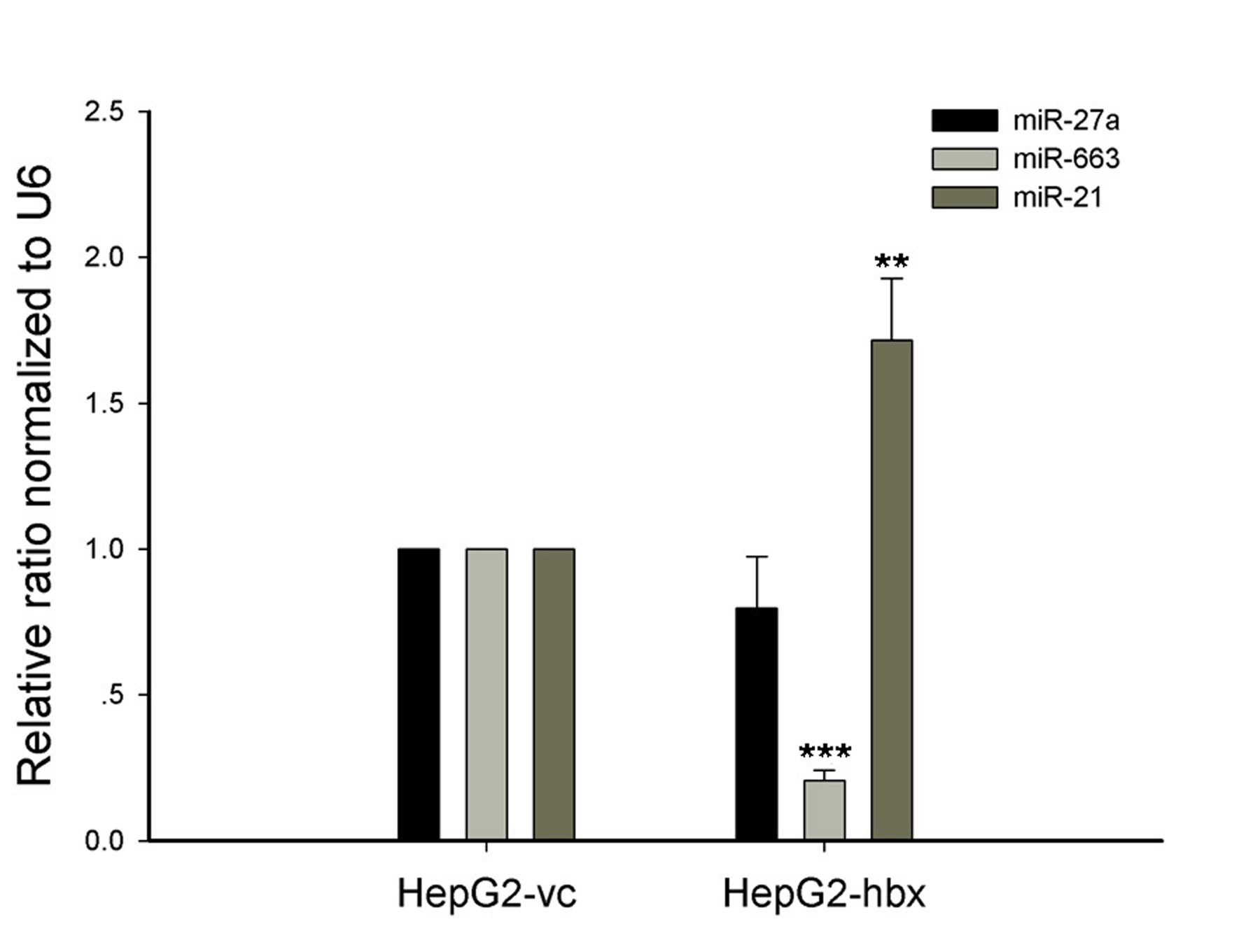

Supplement: Supplementary Figure 2 [file bjc2011190x2.tif]

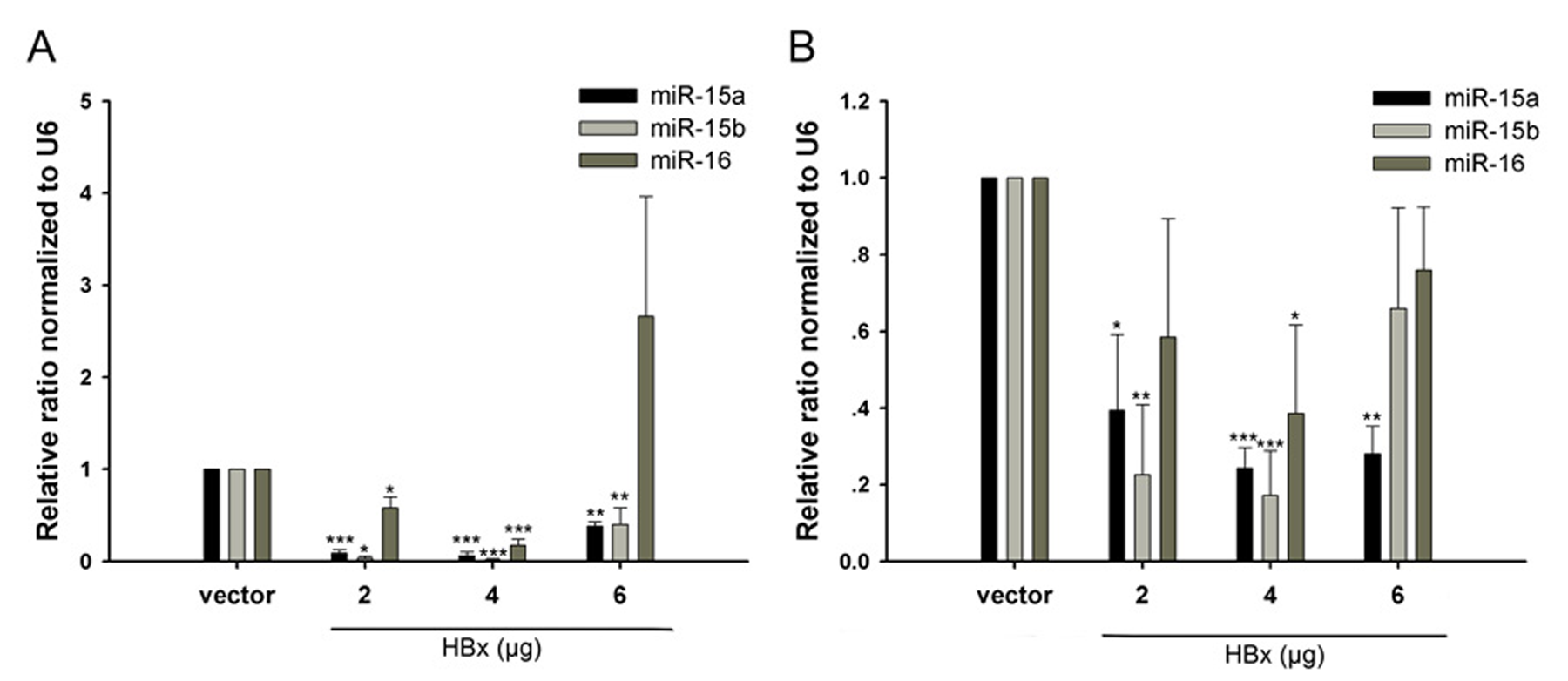

Supplement: Supplementary Figure 3 [file bjc2011190x3.tif]

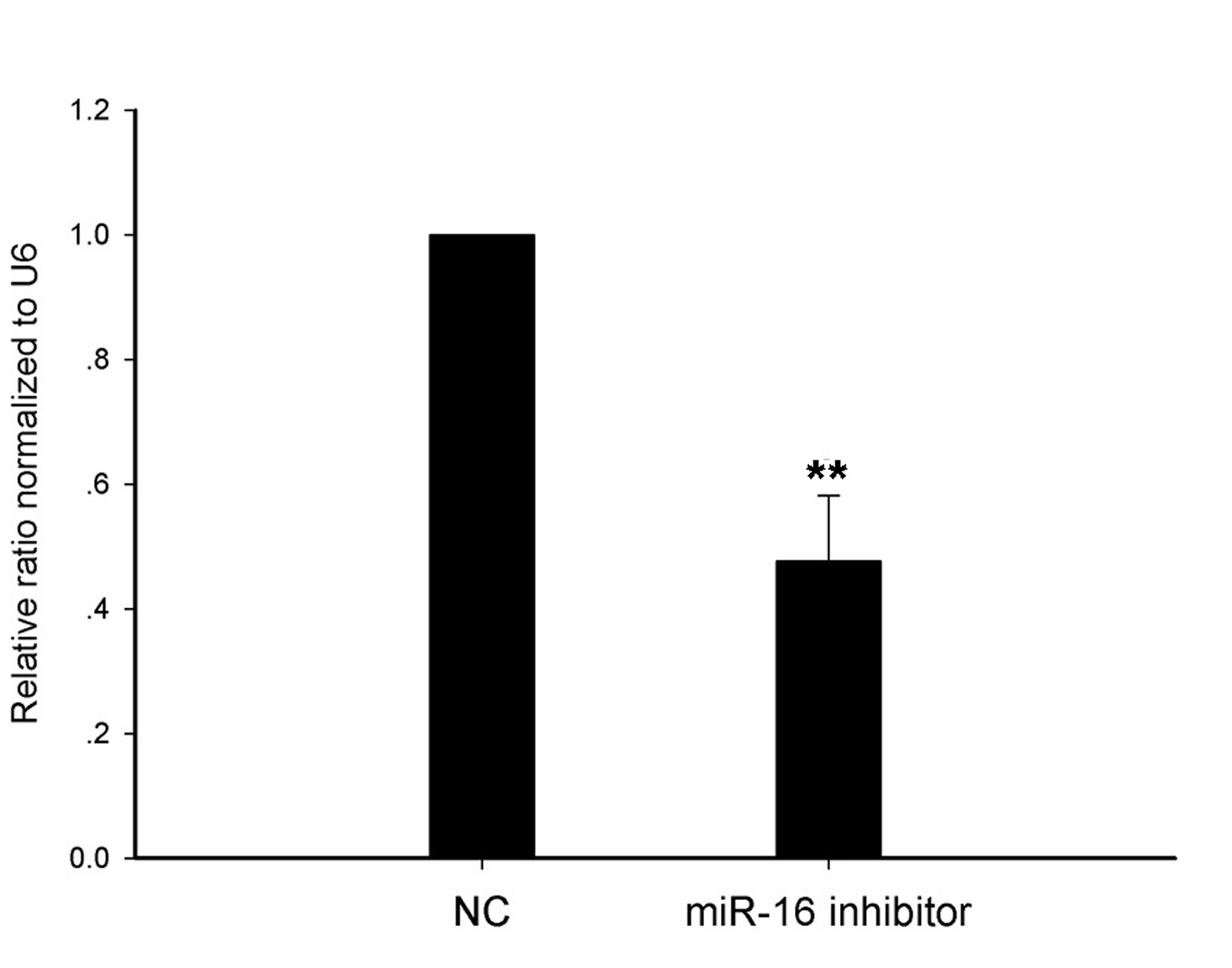

Supplement: Supplementary Figure 4 [file bjc2011190x4.tif]
