# Supplementary material for: Hepatitis B virus X protein downregulates expression of the miR-16 family in malignant hepatocytes in vitro
Source: Br J Cancer. 2011 May 31;105(1):146–53. doi: 10.1038/bjc.2011.190 (PMC3137408; doi:10.1038/bjc.2011.190)
Supplement: Supplementary Figure Legends [file bjc2011190x5.doc]

**Supplementary Figure Legends**

**Supplementary Figure 1. HBx downregulates miR-16 in HepG2 cells.** miR-16 expression was measured with the TaqMan microRNA assay and normalised to the means of RNU48, RNU44, U47 and RNU6B in (A) HepG2 cells stably expressing HBx and (B) HepG2 cells transiently transfected with HBx. The cells were transfected with 2 µg PCDNA3.1-hbx or 2 µg PCDNA3.1 plasmid and were collected for analysis 48 h after each transfection. The data are presented as mean ± SE (***P<0.001, Student’s t-test).

**Supplementary Figure 2. HBx suppressed the expression of miR-27a and miR-663 in HepG2 cells, whereas miR-21 was upregulated.** The results of qRT-PCR showed that HBx significantly deregulated the expression of miR-663 and miR-21 in HepG2 cells, though significant downregulation of miR-27a was not detected. The data are presented as mean ± SE fold (**P < 0.01, ***P<0.001, n=3, Student’s t-test).

**Supplementary Figure 3. miR-16 showed an inverse upregulation in response to increased HBx expression in HepG2 (A) and SK-HEP-1 (B) cells.** Cells were transfected with 2, 4, and 6 µg PCDNA3.1-hbx or 4 µg PCDNA3.1 plasmid and were collected for analysis 48 h after each transfection. The data are presented as mean ± SE fold (*P < 0.05, **P < 0.01, ***P < 0.001; Student’s t-test, NPar tests; n=3).

**Supplementary Figure 4. The expression of miR-16 was reduced in HepG2 cells by a specific inhibitor.** qRT-PCR analysis showed that an miR-16 inhibitor reduced the endogenous abundance of miR-16 by approximately 50% in HepG2 cells (**P < 0.01; Student’s t-test, n=3).
