# Supplementary material for: Hepatitis B virus X protein downregulates expression of the miR-16 family in malignant hepatocytes in vitro
Source: Br J Cancer. 2011 May 31;105(1):146–53. doi: 10.1038/bjc.2011.190 (PMC3137408; doi:10.1038/bjc.2011.190)
Supplement: Supplementary Table 1 [file bjc2011190x6.doc]

| **Name** | **Sense Strand (5'-3')** | **Antisense Strand (5'-3')** |
| --- | --- | --- |
| **miRNA and siRNA Duplexes** |  |  |
| miR-15a | UAGCAGCACAUAAUGGUUUGUG | CAAACCAUUAUGUGCUGCUAUU |
| miR-16 | UAGCAGCACGUAAAUAUUGGCG | CCAAUAUUUACGUGCUGCUAUU |
| siMyc | GGUCAGAGUCUGGAUCACCdTdT | GGUGAUCCAGACUCUGACCdTdT |
| NC | UCACAACCUCCUAGAAAGAGUAGA | UACUCUUUCUAGGAGGUUGUUAUU |
| **miRNA Inhibitors** |  |  |
| anti-miR-16 | CGCCAAUAUUUACGUGCUGCUA |  |
| anti-miR-NC | UUGUACUACACAAAAGUACUG |  |
| **Name** | **Sense Primer (5'-3')** | **Antisense Primer (5'-3')** |
| **Primers for RT-PCR** |  |  |
| β-actin | AAGATGACCCAGATCATGTTTGAG | GCAGCTCGTAGCTCTTCTCCAG |
| HBx | TGTGCACTTCGCTTCACCTC | GAAAAAGTTGCATGGTGCTG |
| **Primers for qRT-PCR** |  |  |
| β-actin | TGGCACCCAGCACAATGAA | CTAAGTCATAGTCCGCCTAGAAGCA |
| c-Myc | GCAGCTGCTTAGACGCTGGA | CGCAGTAGAAATACGGCTGCAC |

**Supplementary Table 1.** Sequences of RNA and DNA Oligonucleotides
