# Supplementary material for: Hepatitis B virus X protein downregulates expression of the miR-16 family in malignant hepatocytes in vitro
Source: Br J Cancer. 2011 May 31;105(1):146–53. doi: 10.1038/bjc.2011.190 (PMC3137408; doi:10.1038/bjc.2011.190)
Supplement: Supplementary Table 2 [file bjc2011190x7.doc]

**Supplementary Table 2.** Result of miRNA microarray analysis

| **microRNA Name** | **hbx- Average** | **vc- Average** | **hbx- Normalized** | **vc- Normalized** | **hbx/vc Ratio** |
| --- | --- | --- | --- | --- | --- |
| hsa-let-7d | 702 | 1108 | 0.05 | 0.12 | **0.45** |
| hsa-miR-105* | 111 | 253 | 0.01 | 0.03 | **0.31** |
| hsa-miR-10b* | 414 | 638 | 0.03 | 0.07 | **0.46** |
| hsa-miR-135a* | 171 | 303 | 0.01 | 0.03 | **0.40** |
| hsa-miR-138-1* | 171 | 470 | 0.01 | 0.05 | **0.26** |
| hsa-miR-144* | 238 | 488 | 0.02 | 0.05 | **0.35** |
| hsa-miR-146b-5p | 220 | 330 | 0.02 | 0.04 | **0.47** |
| hsa-miR-15a | 132 | 198 | 0.01 | 0.02 | **0.47** |
| hsa-miR-15b | 121 | 174 | 0.01 | 0.02 | **0.49** |
| hsa-miR-16 | 269 | 397 | 0.02 | 0.04 | **0.48** |
| hsa-miR-181a | 106 | 207 | 0.01 | 0.02 | **0.36** |
| hsa-miR-181b | 151 | 529 | 0.01 | 0.06 | **0.20** |
| hsa-miR-181d | 109 | 168 | 0.01 | 0.02 | **0.46** |
| hsa-miR-183* | 295 | 436 | 0.02 | 0.05 | **0.48** |
| hsa-miR-184 | 1048 | 1854 | 0.08 | 0.20 | **0.40** |
| hsa-miR-18a* | 108 | 185 | 0.01 | 0.02 | **0.41** |
| hsa-miR-193b | 478 | 304 | 0.05 | 0.02 | **2.22** |
| hsa-miR-19a | 725 | 1446 | 0.06 | 0.16 | **0.36** |
| hsa-miR-200b* | 152 | 232 | 0.01 | 0.03 | **0.47** |
| hsa-miR-203 | 171 | 251 | 0.01 | 0.03 | **0.48** |
| hsa-miR-208b | 144 | 316 | 0.01 | 0.03 | **0.32** |
| hsa-miR-21 | 19230 | 5942 | 1.48 | 0.64 | **2.30** |
| hsa-miR-210 | 557 | 1044 | 0.04 | 0.11 | **0.38** |
| hsa-miR-214 | 883 | 2330 | 0.07 | 0.25 | **0.27** |
| hsa-miR-219-2-3p | 168 | 381 | 0.01 | 0.04 | **0.31** |
| hsa-miR-27a | 639 | 1026 | 0.05 | 0.11 | **0.44** |
| hsa-miR-27b | 594 | 1571 | 0.05 | 0.17 | **0.27** |
| hsa-miR-298 | 486 | 692 | 0.04 | 0.08 | **0.50** |
| hsa-miR-301a | 166 | 240 | 0.01 | 0.03 | **0.49** |
| hsa-miR-302c* | 106 | 218 | 0.01 | 0.02 | **0.35** |
| hsa-miR-30b* | 329 | 1224 | 0.03 | 0.13 | **0.19** |
| hsa-miR-30c-1* | 219 | 560 | 0.02 | 0.06 | **0.28** |
| hsa-miR-323-5p | 142 | 493 | 0.01 | 0.05 | **0.20** |
| hsa-miR-325 | 122 | 216 | 0.01 | 0.02 | **0.40** |
| hsa-miR-338-3p | 111 | 290 | 0.01 | 0.03 | **0.27** |
| hsa-miR-338-5p | 104 | 169 | 0.01 | 0.02 | **0.44** |
| hsa-miR-340 | 308 | 608 | 0.02 | 0.07 | **0.36** |
| hsa-miR-342-5p | 264 | 585 | 0.02 | 0.06 | **0.32** |
| hsa-miR-369-3p | 267 | 469 | 0.02 | 0.05 | **0.40** |
| hsa-miR-370 | 228 | 337 | 0.02 | 0.04 | **0.48** |
| hsa-miR-371-5p | 131 | 320 | 0.01 | 0.03 | **0.29** |
| hsa-miR-373 | 109 | 180 | 0.01 | 0.02 | **0.43** |
| hsa-miR-373* | 2220 | 3958 | 0.17 | 0.43 | **0.40** |
| hsa-miR-374b | 250 | 411 | 0.02 | 0.04 | **0.43** |
| hsa-miR-410 | 193 | 277 | 0.01 | 0.03 | **0.50** |
| hsa-miR-422a | 300 | 444 | 0.02 | 0.05 | **0.48** |
| hsa-miR-423-5p | 1315 | 4014 | 0.10 | 0.44 | **0.23** |
| hsa-miR-485-5p | 153 | 255 | 0.01 | 0.03 | **0.43** |
| hsa-miR-487a | 232 | 349 | 0.02 | 0.04 | **0.47** |
| hsa-miR-489 | 121 | 439 | 0.01 | 0.05 | **0.20** |
| hsa-miR-498 | 903 | 1484 | 0.07 | 0.16 | **0.43** |
| hsa-miR-501-5p | 140 | 272 | 0.01 | 0.03 | **0.37** |
| hsa-miR-509-3p | 179 | 299 | 0.01 | 0.03 | **0.43** |
| hsa-miR-509-5p | 541 | 897 | 0.04 | 0.10 | **0.43** |
| hsa-miR-514 | 104 | 167 | 0.01 | 0.02 | **0.44** |
| hsa-miR-517b | 162 | 264 | 0.01 | 0.03 | **0.44** |
| hsa-miR-524-5p | 119 | 181 | 0.01 | 0.02 | **0.47** |
| hsa-miR-526a;hsa-miR-518d-5p;hsa-miR-518f*;hsa-miR-520c-5p | 125 | 343 | 0.01 | 0.04 | **0.26** |
| hsa-miR-541 | 102 | 211 | 0.01 | 0.02 | **0.34** |
| hsa-miR-542-5p | 158 | 239 | 0.01 | 0.03 | **0.47** |
| hsa-miR-548d-5p | 203 | 310 | 0.02 | 0.03 | **0.47** |
| hsa-miR-552 | 167 | 300 | 0.01 | 0.03 | **0.39** |
| hsa-miR-564 | 172 | 461 | 0.01 | 0.05 | **0.26** |
| hsa-miR-565 | 244 | 539 | 0.02 | 0.06 | **0.32** |
| hsa-miR-575 | 366 | 688 | 0.03 | 0.07 | **0.38** |
| hsa-miR-595 | 146 | 215 | 0.01 | 0.02 | **0.48** |
| hsa-miR-608 | 246 | 588 | 0.02 | 0.06 | **0.30** |
| hsa-miR-610 | 189 | 316 | 0.01 | 0.03 | **0.42** |
| hsa-miR-612 | 208 | 383 | 0.02 | 0.04 | **0.39** |
| hsa-miR-613 | 244 | 390 | 0.02 | 0.04 | **0.44** |
| hsa-miR-629 | 116 | 173 | 0.01 | 0.02 | **0.48** |
| hsa-miR-634 | 127 | 314 | 0.01 | 0.03 | **0.29** |
| hsa-miR-637 | 325 | 675 | 0.03 | 0.07 | **0.34** |
| hsa-miR-648 | 101 | 147 | 0.01 | 0.02 | **0.49** |
| hsa-miR-650 | 214 | 452 | 0.02 | 0.05 | **0.34** |
| hsa-miR-663 | 14949 | 25262 | 1.15 | 2.74 | **0.42** |
| hsa-miR-665 | 169 | 487 | 0.01 | 0.05 | **0.25** |
| hsa-miR-671-5p | 382 | 698 | 0.03 | 0.08 | **0.39** |
| hsa-miR-674 | 817 | 1232 | 0.06 | 0.13 | **0.47** |
| hsa-miR-675 | 470 | 1275 | 0.04 | 0.14 | **0.26** |
| hsa-miR-768-3p | 126 | 184 | 0.01 | 0.02 | **0.49** |
| hsa-miR-768-5p | 160 | 334 | 0.01 | 0.04 | **0.34** |
| hsa-miR-769-3p | 182 | 267 | 0.01 | 0.03 | **0.48** |
| hsa-miR-872 | 161 | 263 | 0.01 | 0.03 | **0.44** |
| hsa-miR-877 | 603 | 1191 | 0.05 | 0.13 | **0.36** |
| hsa-miR-885-3p | 4128 | 13169 | 0.32 | 1.43 | **0.22** |
| hsa-miR-886-5p | 307 | 474 | 0.02 | 0.05 | **0.46** |
| hsa-miR-9* | 113 | 199 | 0.01 | 0.02 | **0.40** |
| hsa-miR-920 | 308 | 488 | 0.02 | 0.05 | **0.45** |
| hsa-miR-922 | 163 | 326 | 0.01 | 0.04 | **0.36** |
| hsa-miR-923 | 5501 | 7965 | 0.42 | 0.86 | **0.49** |
| hsa-miR-92b* | 14400 | 21676 | 1.11 | 2.35 | **0.47** |
| hsa-miR-99b | 227 | 118 | 0.02 | 0.01 | **2.7** |
